# Generic sofosbuvir-based interferon-free direct acting antiviral agents for patients with chronic hepatitis C virus infection: a real-world multicenter observational study

**DOI:** 10.1038/s41598-018-32060-7

**Published:** 2018-09-12

**Authors:** Chen-Hua Liu, Yi-Jie Huang, Sien-Sing Yang, Chung-Hsin Chang, Sheng-Shun Yang, Hsin-Yun Sun, Chun-Jen Liu, Wen-Chun Liu, Tung-Hung Su, Hung-Chih Yang, Chun-Ming Hong, Tai-Chung Tseng, Pei-Jer Chen, Ding-Shinn Chen, Chien-Ching Hung, Jia-Horng Kao

**Affiliations:** 1 Department of Internal Medicine, National Taiwan University Hospital, Taipei, Taiwan; 2 Hepatitis Research Center, National Taiwan University Hospital, Taipei, Taiwan; 3 Department of Internal Medicine, National Taiwan University Hospital, Yun-Lin Branch, Douliou, Taiwan; 4 Department of Internal Medicine, Division of Gastroenterology and Hepatology, Taichung Veterans General Hospital, Taichung, Taiwan; 5 Liver Center, Cathay General Hospital Medical Center, Taipei, Taiwan; 6 School of Medicine, Fu-Jen Catholic University College of Medicine, Taipei, Taiwan; 7 School of Medicine, Chung Shan Medical University, Taichung, Taiwan; 8 Faculty of Medicine, National Yang-Ming University, Taipei, Taiwan; 9 Graduate Institute of Clinical Medicine, National Taiwan University College of Medicine, Taipei, Taiwan; 10 Department of Microbiology, National Taiwan University College of Medicine, Taipei, Taiwan; 11 Department of Traumatology, National Taiwan University Hospital, Taipei, Taiwan; 12 Genomics Research Center, Academia Sinica, Taipei, Taiwan; 13 Department of Parasitology, National Taiwan University College of Medicine, Taipei, Taiwan; 14 Department of Medical Research, China Medical University Hospital, Taichung, Taiwan

## Abstract

Real-world data regarding the effectiveness and safety of generic sofosbuvir (SOF)-based interferon-free direct acting antiviral agents (DAAs) for patients with chronic hepatitis C virus (HCV) infection remain limited. A total of 517 chronic HCV-infected patients receiving 12 or 24 weeks of SOF-based therapies were retrospectively enrolled in 4 academic centers in Taiwan. The rate of sustained virologic response at week 12 off-therapy (SVR_12_) and that of treatment completion were assessed. The baseline characteristics and on-treatment HCV viral kinetics to predict SVR_12_ were analyzed. By evaluable population (EP) analysis, the SVR_12_ rate was 95.4% (95% confidence interval [CI]: 93.2–96.9%). The SVR_12_ was achieved in 29 of 34 patients (85.3%, 95% CI: 69.6–93.6%), 130 of 139 patients (93.5%, 95% CI: 88.2–96.6%), 119 of 124 patients (96.0%, 95% CI: 90.9–98.3%) and 215 of 220 patients (97.7%, 95% CI: 94.8–99.0%) who received SOF in combination with ribavirin (RBV), ledipasvir (LDV), daclatasvir (DCV) and velpatasvir (VEL), respectively. Of 517 patients, 514 (99.4%) completed the scheduled treatment. All 15 patients with true virologic failures were relapsers. Two decompensated cirrhotic patients had on-treatment deaths which were not related to DAAs. All 7 patients who were lost to follow-up had undetectable HCV RNA level at the last visit. The SVR_12_ rates were comparable in terms of baseline patient characteristics and viral decline at week 4 of treatment. In conclusion, generic SOF-based regimens are well tolerated and provide high SVR_12_ rates in patients with chronic HCV infection.

## Introduction

Hepatitis C virus (HCV) infection remains a challenging health problem in the world. It is estimated that approximately 71.1 million people, which account for 1.0% of the world’s population, are HCV carriers^1^. Among patients with chronic HCV infection, about 20% of them will evolve to cirrhosis over a period of 20–30 years. Once cirrhosis is established, the annual rates of developing hepatic decompensation and hepatocellular carcinoma (HCC) are 3–6% and 1–4%, respectively^2,3^. In addition to increasing the risks of liver-related morbidity and mortality, HCV infection is also associated with various extra-hepatic manifestations which further compromised the patients’ health outcome and quality of life^4^. On the other hand, the morbidity and mortality are significantly reduced once these patients achieve sustained virologic response (SVR) by anti-HCV agents^5–9^.

The use of interferon (IFN)-free direct acting antiviral agents (DAAs) has made a paradigm shift and become the standard of care for HCV infection. Sofosbuvir (SOF) is a pyrimidine nucleotide analogue that inhibits the HCV non-structural protein 5B (NS5B) ribonucleic acid (RNA)-dependent RNA polymerase, which is essential for viral replication. After intra-hepatic metabolism to active uridine triphosphate form, the GS-461203, it is incorporated to HCV RNA by NS5B polymerase and acts as the chain terminator^10^. Clinically, SOF is administered once-daily with pangenotypic potency, well tolerability, a high genetic barrier to drug resistance, and low rates of drug-drug interactions (DDIs). Furthermore, SOF can be used in combination with various kinds of NS3/4 A protease inhibitors (PIs), NS5A inhibitors, and/or ribavirin (RBV) to achieve high SVR rates^11–23^. Because of the excellent therapeutic profiles, treatment of HCV by SOF-based regimens is appealing to most health care providers.

Although SOF-based IFN-free DAAs are highly efficacious and well tolerated, many HCV-infected individuals have limited governmental reimbursement or private insurance support for brand-name agents^24–26^. Allowing generic SOF-based DAAs through voluntary or compulsory licensing can scale up the HCV treatment to facilitate more efficient HCV control, particularly for patients in resource-constrained countries. Regarding the effectiveness and safety of generic SOF in combination with ledipasvir (LDV), daclatasvir (DCV), and/or RBV, several reports from China, India, Egypt and Argentina indicated that the SVR rates were >90% and most patients tolerated the treatment well^27–31^. On the basis of these encouraging results, we aimed to evaluate the performance of generic SOF-based DAAs for HCV and factors potentially affecting the treatment response in a multicenter cohort in Taiwan.

## Materials and Methods

### Patients

Between May 2016 and June 2017, HCV-infected patients who received generic SOF-based IFN-free therapies for 12 or 24 weeks at the National Taiwan University Hospital (NTUH), NTUH Yun-Lin Branch, Taichung Veterans General Hospital, and Cathay General Hospital Medical Center were retrospectively enrolled. All patients were aged ≥20 years and had chronic HCV infection, defined as detectable HCV antibody (anti-HCV; Abbott HCV EIA 2.0, Abbott Laboratories, Abbott Park, Illinois, USA) and quantifiable serum HCV RNA (Cobas TaqMan HCV Test v2.0, Roche Diagnostics GmbH, Mannheim, Germany, lower limit of detection [LLOD]: 15 IU/mL) for ≥6 months. Patients were excluded from the study if they had a history of DAA exposure, had estimated glomerular filtration rate (eGFR) <30 mL/min/1.73 m^2^, had active HCC, received antiviral regimens not recommended by American Association for the Study of Liver Diseases/Infectious Diseases Society of America (AASLD/IDSA), European Association for the Study of the Liver (EASL) or Asian Pacific Association for the Study of the Liver (APASL) guidelines, or refused to provide written informed consent^32–34^. The study was approved by the Research Ethics Committee of National Taiwan University Hospital, Taichung Veterans Hospital and Cathay General Hospital and was conducted in accordance with the principles of Declaration of Helsinki and the International Conference on Harmonization for Good Clinical Practice. All patients provided written informed consent before the study.

### Study design

Baseline demographic data, hemogram, serum biochemical profiles (albumin, total bilirubin, aspartate aminotransferase [AST], alanine aminotransferase [ALT], creatinine, eGFR, as calculated by modification of diet in renal disease equation (MDRD), anti-HCV, hepatitis B virus (HBV) surface antigen (Abbott Architect HBsAg qualitative assay, Abbott Laboratories, Abbott Park, Illinois, USA), HCV RNA, HCV genotype (Abbott RealTi*me* HCV Genotype II, Abbott Laboratories, Abbott Park, Illinois, USA) and anti-HIV (Abbott Architect HIV Ag/Ab Combo, Abbott Laboratories, Abbott Park, Illinois, USA) were collected for all patients^35^. The cirrhosis status was determined by liver biopsy, clinical signs of portal hypertension, imaging studies, AST-to-platelet ratio index (ARPI) at a cutoff value of >2.0 or liver stiffness measurement (LSM, FibroScan^®^, Echosens, Paris, France) at a cutoff value of >12.5 kPa when appropriate^36^. In cirrhotic patients, the severity was graded by Child-Pugh score. Baseline serum HBV DNA (Cobas AmpliPrep/Cobas Taqman HBV test v.2.0, Roche Diagnostics GmbH, Mannheim, Germany, LLOD: 20 IU/mL) or HIV RNA (Cobas AmpliPrep/Cobas Taqman HIV-1 test v.2.0, Roche Diagnostics GmbH, Mannheim, Germany, LLOD: 20 copies/mL) level was determined for patients with HBV or HIV coinfection.

Patients received SOF in combination with RBV, LDV, DCV or velpatasvir (VEL) for 12 or 24 weeks. For SOF/RBV regimen, they received a generic version of SOF (400 mg/tablet; Hepcinat^®^, Natco Pharma Ltd., Hyderabad, India; Sofovir^®^, Hetero Corporate Ltd., Hyderabad, India) 1 tablet per day in combination with weight-based RBV (Robatrol^®^, 200 mg capsule, Genovate Biotechnology Co. Ltd., Hsinchu, Taiwan; 1,200 mg per day if the body weight ≥75 kg; 1,000 mg per day if the body weight <75 kg). For SOF/NS5A inhibitor regimens, they received a generic version of fixed-dose combination SOF/LDV (400/90 mg tablet; Hepcinat-LP^®^, Natco Pharma Ltd., Hyderabad, India; Ledifos^®^, Hetero Corporate Ltd., Hyderabad, India), SOF/DCV (400/60 mg tablet; Darvoni^®^, Beacon Pharmaceuticals Ltd. Mymensingh, Bangladesh) or SOF/VEL, (400/100 mg tablet; Velpanat^®^, Natco Pharma Ltd., Hyderabad, India; Velasof^®^, Hetero Corporate Ltd., Hyderabad, India; MyHep All^®^, Mylan Laboratories Ltd., Hyderabad, India; Sofosvel^®^, Beacon Pharmaceuticals Ltd. Mymensingh, Bangladesh) 1 tablet per day with or without weight-based RBV.

### Effectiveness

Patients received on-treatment serum HCV RNA testing at weeks 4 and 12. For patients receiving 12 and 24 weeks of treatment, serum HCV RNA levels were assessed at treatment weeks 12 and 24 to determine antiviral responses at end-of-treatment (EOT). Furthermore, they received off-therapy serum HCV RNA testing at week 12 to assess SVR_12_. If patients prematurely discontinued treatment, the antiviral response at EOT was assessed at the time of last visit. Patients were considered failure to achieve SVR_12_ if they lacked SVR_12_ data. We adopted two different endpoints for effectiveness: the evaluable population (EP) which assessed the SVR_12_ for patients who received at least one dosage of treatment were included in the analysis, and the per-protocol population (PP) which assessed the SVR_12_ by excluding non-SVR_12_ patients due to non-virologic failure.

### Safety

The rate of treatment completion was assessed for each regimen. The reasons for patients who were lost to follow-up were recorded by the treating physicians. In patients who were seropositive for HBsAg, serum HBV DNA levels were evaluated after the initiation of DAA treatment. HBV reactivation was defined as the presence of HBV DNA level ≥LLOD in patients with baseline HBV DNA level <LLOD, or increase of HBV DNA level >1 log_10_ IU/mL in patients with baseline HBV DNA level ≥LLOD^37^. HBV-associated hepatitis was defined as HBV reactivation and hepatitis flare presenting with ALT increase ≥3 times baseline and >100 U/L^38^.

### Statistical analysis

All analyses were performed using Statistical Program for Social Sciences (SPSS Statistics Version 23.0, IBM Corp., Armonk, New York, USA). The baseline characteristics were shown in median (range) and percentages when appropriate. The viral responses during and after treatment were shown in number and percentages with 95% confidence interval (CI). The stratified analysis of SVR_12_ by baseline characteristics and week 4 viral decline were assessed and shown in percentages with 95% CI.

## Results

### Patient characteristics

**Figure 1:**
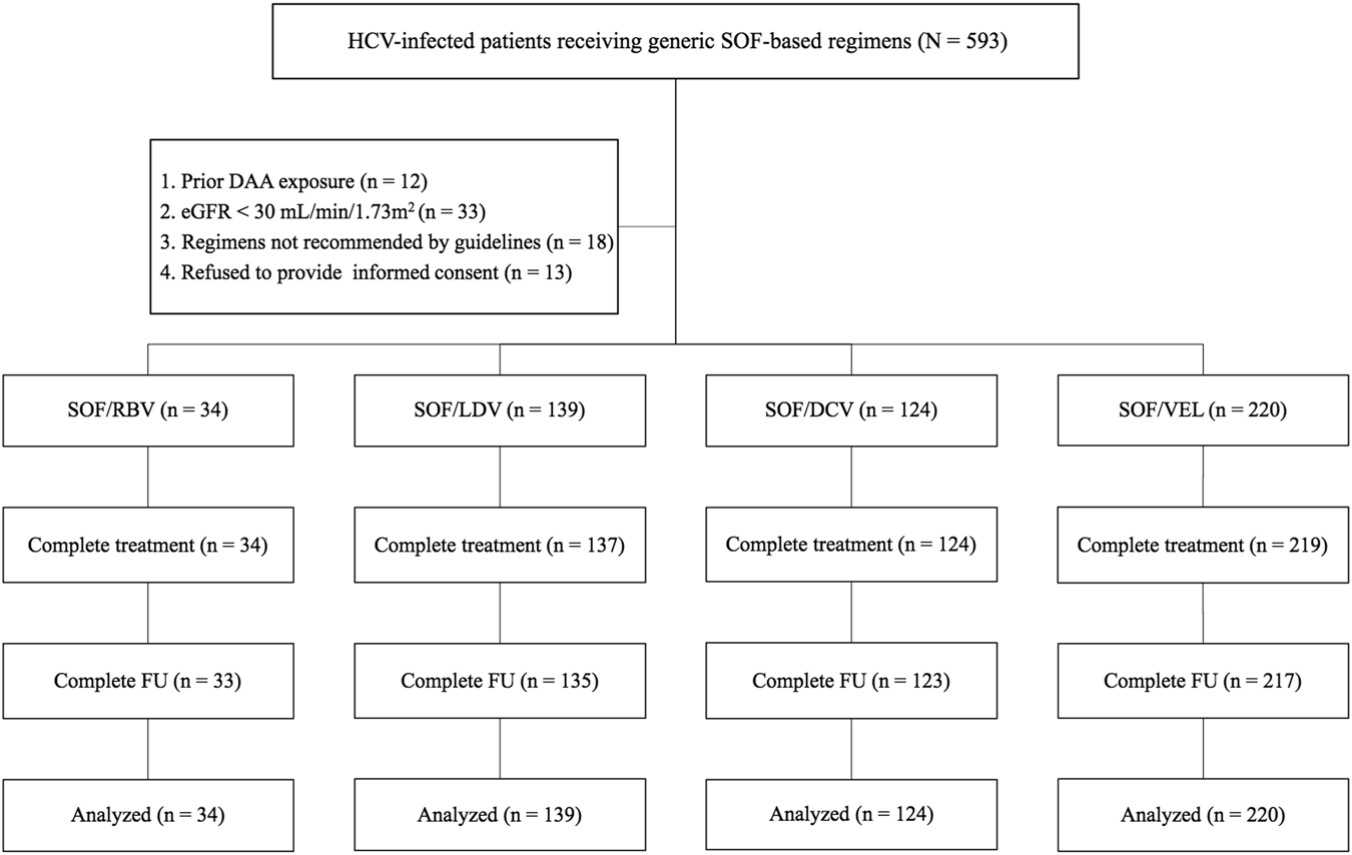
Study flow.

Of 593 HCV-infected patients receiving SOF-based IFN-free DAAs, 76 were excluded from the study because of prior DAA exposure, eGFR < 30 mL/min/1.73 m^2^, receiving antiviral regimens not recommended by guidelines, or refusal to provide informed consent. The remaining 517 patients were eligible for the analysis (Fig. 1). Table 1 shows the baseline patient characteristics. Thirty-four (6.6%), 139 (26.9%), 124 (24.0%) and 220 (42.6%) patients receiving SOF in combination with RBV, LDV, DCV and VEL, respectively. Patients receiving SOF/VEL tended to be younger, have a higher percentage of HIV-coinfected patients and a lower percentage of cirrhosis, compared to those receiving other SOF-based regimens. Among patients receiving SOF in combination with NS5A inhibitor, those receiving SOF/LDV tended to have a higher percentage of combining RBV usage, compared to those receiving SOF/DCV or SOF/VEL. All patients receiving SOF/RBV were infected with HCV genotype 2 (HCV-2) and were treated for 12 weeks, whereas all receiving SOF/LDV were infected with HCV-1a, HCV-1b or HCV-6. Sixty-nine (55.6%), one (0.8%), two (1.6%) and two (1.6%) receiving SOF/DCV had HCV-2, HCV-3, HCV-6, and mixed HCV genotype 1b + 2 and 2 + 6 infections. Patients receiving SOF/VEL had HCV-1 to 6 infection. One HCV viremic patient with indeterminate genotyping received SOF/VEL. No patients receiving SOF/RBV had decompensated cirrhosis. Among patients with HBV or HIV coinfection, 60–75% and 94.4–100% of them had baseline undetectable HBV DNA or HIV RNA levels.

**Table 1:** Baseline patient characteristics.

Characteristics*	SOF/RBV (N = 34)	SOF/LDV (N = 139)	SOF/DCV (N = 124)	SOF/VEL (N = 220)
Age, year, median (range)	58 (25–81)	61 (29–85)	61 (27–86)	57 (25–85)
Age ≥ 55 years	25 (73.5)	101 (72.7)	91 (73.4)	124 (56.4)
Male	11 (32.4)	68 (48.9)	45 (36.3)	128 (58.2)
IFN-based treatment-naive	25 (73.5)	86 (61.9)	87 (70.2)	172 (78.2)
HBV coinfection	4 (11.8)	10 (7.2)	6 (4.8)	21 (9.5)
HBV DNA < LLOD in HBV coinfection^†^	3 (75.0)	6 (60.0)	4 (75.0)	15 (71.4)
HIV coinfection	1 (2.9)	4 (2.9)	2 (1.6)	54 (24.5)
HIV RNA < LLOD in HIV coinfection^†^	1 (100)	4 (100)	2 (100)	51 (94.4)
Prior history of HCC	2 (5.9)	11 (7.9)	20 (16.1)	12 (5.5)
Scheduled DAA treatment				
12 weeks	34 (100)	133 (95.7)	104 (83.9)	217 (98.6)
24 weeks	0 (0)	6 (4.3)	20 (16.1)	3 (1.4)
RBV usage	34 (100)	50 (36.0)	5 (4.0)	14 (6.4)
BMI ≥ 25 kg/m^2^	18 (52.9)	46 (33.1)	44 (35.5)	76 (34.5)
Hemoglobin, g/dL, median (range)	14.2 (10.3–16.8)	13.4 (6.4–17.6)	13.2 (8.4–17.5)	14.1 (8.3–17.6)
White cell count, 10^9^ cells/L, median (range)	5.4 (3.5–13.7)	5.2 (1.7–12.5)	5.0 (1.8–15.9)	5.2 (2.2–15.9)
Platelet count, 10^9^ cells/L, median (range)	135 (33–289)	145 (22–433)	153 (39–164)	175 (28–433)
Albumin, g/dL, median (range)	4.2 (3.8–4.8)	4.1 (2.3–5.2)	4.2 (2.8–5.2)	4.3 (3.9–5.4)
Total bilirubin, mg/dL, median (range)	0.9 (0.3–2.2)	0.9 (0.3–8.7)	0.8 (0.3–4.3)	0.8 (0.3–4.6)
AST, ULN, median (range)	1.7 (0.5–10.0)	1.6 (0.5–7.9)	1.9 (0.5–9.7)	1.6 (0.4–12.8)
ALT, ULN, median (range)	2.5 (0.7–11.2)	1.7 (0.3–14.4)	1.9 (0.5–13.4)	1.9 (0.2–13.4)
ALT > 2X ULN	19 (55.9)	60 (43.2)	60 (48.4)	107 (48.6)
Creatinine, mg/dL, median (range)	0.8 (0.6–1.6)	0.9 (0.5–2.0)	0.8 (0.4–2.3)	0.8 (0.5–1.7)
eGFR, mL/min/1.73 m^2^, median (range)^‡^	83.7 (37.6–135.0)	84.1 (32.8–150.4)	89.0 (37.0–178.8)	93.1 (37.0–198.3)
eGFR < 60 mL/min/1.73 m^2^‡	6 (17.6)	24 (17.3)	22 (17.7)	37 (16.8)
HCV RNA, log_10_ IU/mL, median (range)	6.05 (4.46–7.15)	6.08 (2.85–7.70)	5.91 (1.85–7.59)	6.19 (1.83–7.70)
HCV RNA > 6,000,000 IU/mL	4 (11.8)	17 (12.2)	11 (8.9)	39 (17.7)
HCV genotype				
1a	0 (0)	6 (4.3)	3 (2.4)	19 (8.6)
1b	0 (0)	125 (89.9)	47 (37.9)	96 (43.6)
1^§^	0 (0)	0 (0)	0 (0)	1 (0.5)
2	34 (100)	0 (0)	69 (55.6)	82 (37.3)
3	0 (0)	0 (0)	1 (0.8)	7 (3.2)
4	0 (0)	0 (0)	0 (0)	2 (0.9)
6	0 (0)	8 (5.8)	2 (1.6)	12 (5.5)
Mixed^¶^	0 (0)	0 (0)	2 (1.6)	0 (0)
Untypable	0 (0)	0 (0)	0 (0)	1 (0.5)
Cirrhosis				
Absent	17 (50.0)	75 (54.0)	74 (59.7)	164 (74.5)
Present	17 (50.0)	64 (46.0)	50 (40.3)	56 (25.5)
Child-Pugh A	17 (50.0)	44 (31.7)	39 (31.5)	44 (20.0)
Child-Pugh B and C	0 (0)	20 (14.4)	11 (8.9)	12 (5.5)

SOF: sofosbuvir; RBV: ribavirin; LDV: ledipasvir; DCV: daclatasvir; VEL: velpatasvir; IFN: interferon; HBV: hepatitis B virus; HIV: human immunodeficiency virus; HCC: hepatocellular carcinoma; DAA: direct acting antiviral agent; BMI: body mass index; AST: aspartate aminotransferase; ALT: alanine aminotransferase; ULN: upper limit of normal; eGFR: estimated glomerular filtration rate.

*Values are numbers (percentages) unless otherwise indicated.

^†^HBV DNA LLOD: 20 IU/mL; HIV RNA LLOD: 20 copies/mL.

^‡^eGFR was calculated by MDRD equation.

^§^Failed subtyping for major genotyping.

^¶^SOF/DCV arm: one patient with genotype 1b + 2 infection, and one patient with genotype 2 + 6 infection.

### Effectiveness

At week 4 of treatment, 30 of 34 (88.2%; 95% CI: 73.4–95.3%), 125 of 138 (90.6%; 95% CI: 84.6–94.4%), 112 of 114 (90.3%; 95% CI: 83.8–94.4%) and 196 of 220 (89.1%; 95% CI: 84.3–92.6%) patient receiving SOF in combination with RBV, LDV, DCV and VEL had undetectable serum HCV RNA level, respectively. Overall, 463 of 516 (89.7%; 95% CI: 86.8–92.1%) patients and 516 of 517 (99.8%; 95% CI: 98.9–100%) patients had undetectable serum HCV RNA levels at week 4 of treatment and at the end-of-treatment. One Child-Pugh C cirrhotic patient receiving SOF/LDV plus RBV died at treatment day 12 and the HCV RNA level at treatment week 1 was 1,047 IU/mL. The overall SVR_12_ rates were 95.4% (493 of 517 patients; 95% CI: 93.2–96.9%) by EP analysis, and 97.1% (493 of 508 patients; 95% CI: 95.2–98.2%) by PP analysis. The SVR_12_ rates for patients receiving SOF in combination with RBV, LDV, DCV, and VEL were 85.3% (95% CI: 69.9–93.6%), 93.5% (95% CI: 88.2–96.6%), 96.0% (95% CI: 90.9–98.3%) and 97.7% (95% CI: 94.8–99.0%) by EP analysis, and were 87.9% (95% CI: 72.7–95.2%), 96.3% (95% CI: 91.6–98.4%), 96.8% (95% CI: 91.9–98.7%) and 99.1% (95% CI: 96.7–99.8%) by PP analysis, respectively (Table 2).

**Table 2:** Virologic responses.

HCV RNA < LLOD*	Overall (N = 517)		SOF/RBV (N = 34)		SOF/LDV (N = 139)		SOF/DCV (N = 124)		SOF/VEL (N = 220)	
	n/N (%)	95% CI	n/N (%)	95% CI	n/N (%)	95% CI	n/N (%)	95% CI	n/N (%)	95% CI
During treatment										
Week 4	463/516 (89.7)	86.8–92.1	30/34 (88.2)	73.4–95.3	125/138 (90.6)	84.6–94.4	112/124 (90.3)	83.8–94.4	196/220 (89.1)	84.3–92.6
Week 12	514/514 (100)	99.3–100	34/34 (100)	89.9–100	137/137 (100)	97.3–100	124/124 (100)	97.0–100	219/219 (100)	98.3–100
Week 24	29/29 (100)	88.3–100	NA	NA	6/6 (100)	61.0–100	20/20 (100)	83.9–100	3/3 (100)	43.9–100
End of treatment^†^	516/517 (99.8)	98.9–100	34/34 (100)	89.9–100	138/139 (99.3)	96.0–99.9	124/124 (100)	97.0–100	220/220 (100)	98.3–100
After treatment										
SVR_12_ (EP)^‡^	493/517 (95.4)	93.2–96.9	29/34 (85.3)	69.9–93.6	130/139 (93.5)	88.2–96.6	119/124 (96.0)	90.9–98.3	215/220 (97.7)	94.8–99.0
SVR_12_ (PP)^§^	493/508 (97.1)	95.2–98.2	29/33 (87.9)	72.7–95.2	130/135 (96.3)	91.6–98.4	119/123 (96.8)	91.9–98.7	215/217 (99.1)	96.7–99.8
Reason for non-SVR_12_, n										
Relapse	15		4		5		4		2	
Lost to follow-up	9		1		4		1		3	
During treatment	3		0		2		0		1	
After treatment	6		1		2		1		2	

*HCV RNA LLOD: 15 IU/mL.

^†^Defined as the HCV RNA level at the time point of on-treatment last visit.

^‡^Evaluable population (EP): patients who received at least one dosage of treatment were included in the analysis.

^§^Per-protocol population (PP): patients with non-virologic failure were excluded from the analysis.

Among patients who failed to achieved SVR_12_, 15 (2.9%) were relapsers and 9 (1.7%) were lost to follow-up. Among the 15 relapsers, 7 (46.7%) were male, 9 (60%) were treatment-naïve, and 10 (66.7%) had cirrhosis. Eight of the 9 (88.9%) patients who were lost to follow-up had HCV RNA level < LLOD at the last visit (Table 3).

**Table 3:** Summary of patients who failed to achieve SVR_12_.

Patient No.	Age	Sex	IFN experience	HCV RNA, log10 IU/mL	HCV GT	Cirrhosis	Child-Pugh	DAA regimen	Scheduled Tx, week	Actual Tx, week	Time point of LTFU	HCV RNA at the last visit, log10 IU/mL	Others
**Relapse**													
1	75	M	Naïve	5.80	2	Present	A	SOF/RBV	12	12	—	3.79	—
2	57	M	Naïve	6.44	2	Absent	—	SOF/RBV	12	12	—	4.05	—
3	43	M	Experienced	6.79	2	Absent	—	SOF/RBV	12	12	—	6.15	—
4	60	F	Experienced	6.26	2	Present	A	SOF/RBV	12	12	—	6.53	—
5	46	F	Naïve	6.46	1b	Absent	—	SOF/LDV	12	12	—	6.47	—
6	41	F	Experienced	6.26	1b	Present	A	SOF/LDV/RBV	12	12	—	6.52	—
7	58	M	Experienced	6.82	1b	Absent	—	SOF/LDV	12	12	—	6.56	—
8	85	F	Naïve	6.06	1b	Present	C	SOF/LDV/RBV	12	12	—	6.37	—
9	78	F	Naïve	6.12	1b	Present	B	SOF/LDV/RBV	12	12	—	5.90	—
10	51	F	Naive	6.55	1b	Present	A	SOF/DCV	12	12	—	6.23	—
11	76	M	Naïve	3.79	2	Present	A	SOF/DCV	12	12	—	5.52	—
12	84	F	Experienced	5.75	1b	Present	A	SOF/DCV	24	24	—	6.87	—
13	65	F	Experienced	6.00	1b	Present	A	SOF/DCV	24	24	—	6.33	—
14	75	M	Naïve	5.80	1a	Present	A	SOF/VEL	12	12	—	3.79	—
15	57	M	Naïve	6.44	1a	Absent	—	SOF/VEL	12	12	—	4.05	HIV coinfection
**LTFU**													
1	56	F	Naïve	6.63	2	Absent	—	SOF/RBV	12	12	SVR_8_	<LLOD	Declined outpatient FU
2	68	M	Experienced	6.59	1b	Present	C	SOF/LDV/RBV	12	1	Tx week 2	3.02	Expired at treatment day 12 due to SBP
3	68	M	Naïve	5.23	1b	Absent	—	SOF/LDV	12	12	SVR_4_	<LLOD	Declined outpatient FU
4	65	F	Experienced	6.37	1b	Present	A	SOF/LDV	24	24	SVR_4_	<LLOD	Declined outpatient FU
5	56	M	Naïve	5.63	1b	Present	B	SOF/LDV/RBV	12	10	Tx week 12	<LLOD	Expired at treatment week 11 due to SBP
6	53	F	Naïve	6.60	2	Absent	—	SOF/DCV	12	12	SVR_8_	<LLOD	Declined outpatient FU
7	65	F	Naïve	1.83	2	Present	A	SOF/VEL	12	8	Tx week 12	<LLOD	Declined outpatient FU
8	28	M	Naïve	6.85	1b	Absent	—	SOF/VEL	12	12	SVR_12_	<LLOD	Declined outpatient FU, HIV coinfection
9	80	M	Naïve	5.67	2	Present	A	SOF/VEL	12	12	SVR_4_	<LLOD	Declined outpatient FU

GT: genotype, Tx: treatment, LTFU: lost-to follow-up, SBP: spontaneous bacterial peritonitis.

**Figure 2:**
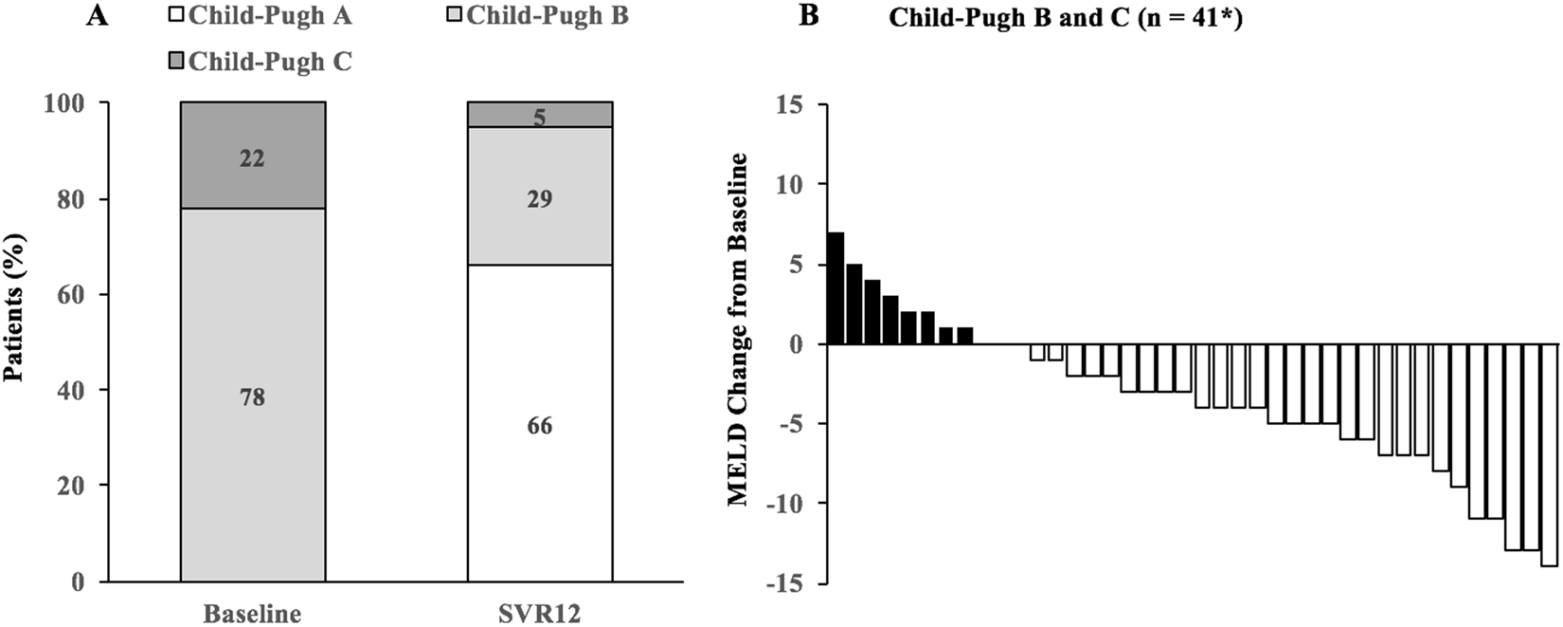
(**A**) Child-Pugh class shift in cirrhotic patients with baseline Child-Pugh B and C, (**B**) Changes of MELD scores from baseline. *Two patients who died during treatment were excluded from the analysis. MELD: model for end-stage liver disease.

Among the 41 decompensated cirrhotic patients with available baseline and end of follow-up data, 32 (78%) and 9 (22%) of them had baseline Child-Pugh B and C. At the end of follow-up, 27 (66%), 12 (29%) and 2 (5%) of them had Child-Pugh A, B, and C, respectively (Fig. 2A). Regarding the scores for model for end-stage liver disease (MELD), 8 (20%), 3 (7%) and 30 (73%) of them had worsened, stable, and improved scores at the end of follow-up, as compared to the baseline status (Fig. 2B).

### Stratified analysis of baseline characteristics predictive of SVR_12_

Table 4 shows the stratified SVR_12_ rates of SOF-based DAA regimens by baseline characteristics and week 4 treatment response. The SVR_12_ rates were comparable with regard to age at a cut-off value of 55 years, sex, prior IFN exposure, HBV or HIV coinfection, prior history of HCC, scheduled 12 or 24 weeks of treatment, use of RBV, BMI at a cut-off value of 25 kg/m^2^, ALT quotient at a cut-off of 2, eGFR at a cut-off value of 60 mL/min/1.73 m^2^, baseline HCV viral load at a cut-off value of 6,000,000 IU/mL, HCV genotype, cirrhosis and week 4 viral decline in patients receiving SOF-based regimens. The SVR_12_ rates for compensated cirrhotic and decompensated cirrhotic patients were 95.5% (95% CI: 84.9–98.7%) and 80.0% (95% CI: 58.4–91.9%) receiving SOF/LDV, 89.7% (95% CI: 76.4–95.9%) and 100% (95% CI: 74.1–100%) receiving SOF/DCV, and 93.2% (95% CI: 81.8–97.7%) and 100% (95% CI: 75.8–100%) receiving SOF/VEL. In addition, the SVR_12_ was achieved in 42 of 45 (93.3%; 95% CI: 82.1–97.7%) patients with a prior history of HCC and in 451 of 472 (95.6%; 95% CI: 93.3–97.1%) patients without a prior history of HCC.

**Table 4:** Sustained virologic response at week 12 off therapy (SVR_12_) according to baseline patient characteristics and HCV viral decline at week 4 of treatment.

Characteristics	SOF/RBV (N = 34)			LDV/SOF (N = 139)			DCV/SOF (N = 124)			VEL/SOF (N = 220)		
	Patient No.	SVR_12_ (%)	95% CI	Patient No.	SVR_12_ (%)	95% CI	Patient No.	SVR_12_ (%)	95% CI	Patient No.	SVR_12_ (%)	95% CI
Age, years												
<55	9	88.9	56.5–98.0	38	94.7	82.7–98.6	33	93.9	80.4–98.3	96	99.0	94.3–99.8
≥55	25	84.0	65.4–93.6	101	93.1	86.4–96.6	91	96.7	90.8–98.9	124	96.8	92.0–98.7
Sex												
Male	11	72.7	43.4–90.3	68	94.1	85.8–97.7	45	97.8	88.4–99.6	128	96.9	92.3–98.8
Female	23	91.3	73.2–97.6	71	93.0	84.6–97.0	79	94.9	87.7–98.0	92	98.9	94.1–99.8
Prior IFN-based treatment												
Naïve	25	88.0	70.0–95.8	86	93.0	85.6–96.8	87	96.6	90.3–98.8	172	97.1	93.4–98.8
Experienced	9	77.8	45.3–93.7	53	94.3	84.6–98.1	37	94.6	82.3–98.5	48	100	96.3–100
HBV coinfection												
Absent	30	83.3	66.4–92.7	129	93.0	87.3–96.3	118	95.8	90.5–98.2	199	97.5	94.3–98.9
Present	4	100	51.0–100	10	100	72.3–100	6	100	61.0–100	21	100	84.5–100
HIV coinfection												
Absent	33	84.9	69.1–93.4	135	93.3	87.8–96.5	122	95.9	90.8–98.2	166	98.2	94.8–99.4
Present	1	100	20.7–100	4	100	51.0–100	2	100	34.2–100	54	96.3	87.5–99.0
Prior history of HCC												
Yes	2	100	34.2–100	11	81.8	52.3–94.9	20	100	83.9–100	12	91.7	64.6–98.5
No	32	84.4	68.3–93.1	128	94.5	89.1–97.3	104	95.2	89.2–97.9	208	98.1	95.2–99.3
Scheduled DAA treatment, week												
12	34	85.3	69.6–93.6	133	94.0	88.6–96.9	104	97.1	91.9–99.0	217	97.7	94.7–99.0
24	0	NA	NA	6	83.3	43.7–97.0	20	90.0	69.9–97.2	3	100	43.9–100
RBV usage												
No	0	NA	NA	89	95.5	89.0–98.2	119	95.8	90.5–98.2	206	97.6	94.4–99.0
Yes	34	85.3	69.6–93.6	50	90.0	78.6–95.7	5	100	56.6–100	14	100	78.5–100
BMI, kg/m^2^												
<25	16	75.0	50.5–89.8	93	93.6	86.6–97.0	80	96.3	89.6–98.7	144	97.9	94.1–99.3
≥ 25	18	94.4	74.2–99.0	46	93.5	82.5–97.8	44	95.5	84.9–98.7	76	97.4	90.9–99.3
ALT > 2X ULN												
No	15	93.3	70.2–98.8	79	92.4	84.4–96.5	64	93.8	85.0–97.5	113	98.2	93.8–99.5
Yes	19	79.0	56.7–91.5	60	95.0	86.3–98.3	60	98.3	91.1–99.7	107	97.2	92.1–99.0
eGFR, mL/min/1.73 m^2^												
<60	6	66.7	30.0–90.3	24	91.7	74.2–97.7	22	90.9	72.2–97.5	37	94.6	82.3–98.5
≥60	28	89.3	72.8–96.3	115	93.9	88.0–97.0	102	97.1	91.7–99.0	183	98.4	95.3–99.4
HCV RNA, IU/mL												
<6,000,000	30	86.7	70.3–94.7	122	93.4	87.6–96.6	113	95.6	90.1–98.1	181	97.8	94.5–99.1
≥6,000,000	4	75.0	30.1–95.4	17	94.1	73.0–99.0	11	100	74.1–100	39	97.4	86.8–99.6
HCV genotype												
1a	0	NA	NA	6	100	61.0–100	3	100	43.9–100	19	89.5	68.6–97.1
1b	0	NA	NA	125	92.8	86.9–96.2	47	93.6	82.8–97.8	96	99.0	94.3–99.8
1	0	NA	NA	0	NA	NA	0	NA	NA	1	100	20.7–100
2	34	85.3	69.6–93.6	0	NA	NA	69	97.1	90.0–99.2	82	97.6	91.5–99.3
3	0	NA	NA	0	NA	NA	1	100	20.7–100	7	100	64.6–100
4	0	NA	NA	0	NA	NA	0	NA	NA	2	100	34.2–100
6	0	NA	NA	8	100	67.6–100	2	100	34.2–100	12	100	75.8–100
Mixed	0	NA	NA	0	NA	NA	2	100	34.2–100	0	NA	NA
Untypable	0	NA	NA	0	NA	NA	0	NA	NA	1	100	20.7–100
Cirrhosis												
Absent	17	82.4	60.0–93.8	75	96.0	88.9–98.6	74	98.7	92.7–99.8	164	98.8	95.7–99.7
Present	17	88.2	65.7–96.7	64	90.6	81.0–95.6	50	92.0	81.2–96.9	56	94.6	85.4–98.2
Child-Pugh A	17	88.2	65.7–96.7	44	95.5	84.9–98.7	39	89.7	76.4–95.9	44	93.2	81.8–97.7
Child-Pugh B and C	0	NA	NA	20	80.0	58.4–91.9	11	100	74.1–100	12	100	75.8–100
Week 4 HCV RNA < LLOD												
No	4	75.0	30.1–95.4	13	92.3	66.7–98.6	12	100	75.8–100	24	100	86.2–100
Yes	30	86.7	70.3–94.7	125	94.4	88.9–97.3	112	95.5	90.0–98.1	196	97.5	94.2–98.9

NA: not assessed.

### Safety

Five hundred fourteen of 517 (99.4%) patients completed the scheduled treatment. One Child-Pugh C and one Child-Pugh B cirrhotic patients receiving SOF/LDV plus RBV died at treatment day 12 and week 11 due to spontaneous bacterial peritonitis, respectively. One Child-Pugh A cirrhotic patient receiving SOF/VEL declined treatment after week 8 and another 6 patients declined off-therapy follow-ups. The reasons for lost to follow-up in the 7 patients were not related to DAA treatment (Table 3). Among the 41 HCV-infected patients with HBV coinfection, 9 (22.0%) also had HIV coinfection. All the 9 patients received tenofovir (TDF)-based antiretroviral agents (ARTs) and none had baseline detectable serum HBV DNA level or had HBV reactivation after DAA treatment. Nineteen of 32 (59.4%) HBV/HCV-coinfected patients without HIV infection had baseline undetectable serum HBV DNA level; 13 (40.6%) had detectable serum HBV DNA levels (range: 25 to 1,820 IU/mL). All the 32 patients did not receive oral nucleos(t)ide analogues or peginterferon for HBV prior to DAA treatment. Eighteen (56.3%) patients met the virologic criteria for HBV reactivation after the initiation of DAA treatment. One (3.1%) patient receiving SOF/LDV developed HBV-associated hepatitis at week 8 of treatment. The baseline HBV DNA level was 1,540 IU/mL, which peaked to 54,200 IU/mL at week 8 of treatment. The ALT level was 192 U/L and the serum HCV RNA level was <LLOD at the time of HBV reactivation. No concomitant serum total bilirubin level elevation or signs of hepatic decompensation was present. The patient received entecavir at week 9 and the ALT level normalized after 7 weeks of treatment.

## Discussion

Compared to PI-containing HCV DAA regimens such as paritaprevir/ritonavir, ombitasvir plus dasabuvir (PrOD), elbasvir/grazoprevir (EBR/GZR), or daclatasvir/asunaprevir (DCV/ASV), PI-free SOF-based regimens have relatively lower pill burden, broader genotype/subtype coverage, fewer drug-drug interactions (DDIs), and can be applied to patients with decompensated cirrhosis^11–19,39^. Although the newly developed glecaprevir/pibrentasvir (GLE/PIB) and voxilaprevir (VOX)/SOF/VEL are potent regimens with pangenotypic activity, they are contraindicated for decompensated cirrhotic patients. Therefore, PI-free SOF-based regimens are appealing choices to health care providers for treating HCV infection.

Our study showed that the overall SVR_12_ rate in patients receiving generic SOF in combination with RBV or NS5A inhibitors was excellent (95.4%) and was comparable to the response rates in patients receiving brand-name agents^12–23^. The per-protocol SVR_12_ rate was 97.1% after excluding patients with non-virologic failure. Regarding safety, >99% of our patients completed the scheduled treatment. Only 2 decompensated cirrhotic patients prematurely discontinued treatment due to spontaneous bacterial peritonitis, which were considered not related to DAA usage.

In our patients receiving SOF/RBV, all were infected with HCV-2, which reflected the potential suboptimal response rates in patients infected with other genotypes. The SVR_12_ rate for SOF/RBV in our study was 85.3%, which was comparable to the response rates in clinical trials^12,13^. Although there were no statistical differences for SVR_12_ rates by baseline patient characteristics, our data were in line with VALENCE study that the CIs of the response rates varied widely, probably due to the small case numbers in both studies^13^. In HCV-2 cirrhotic patients receiving SOF/RBV for 12 weeks, the SVR_12_ rates in FUSION and VALENCE trials as well as Western real-world practice were 60–82%^12,13,40^. However, the SVR_12_ rate in our HCV-2 cirrhotic patients was higher (88.2%) than Western reports and was comparable to the response rates in East-Asian trials^41–43^. The factors attributed to the superior response rates in East-Asian patients to Western patients are still unknown. Furthermore, whether extending the treatment to 16 weeks could achieve better response rates in our HCV-2 cirrhotic patients needs further evaluation^12,44^.

The SVR_12_ rate of our patients receiving generic SOF/LDV-based therapies was 93.5%, which was comparable to the response rates in patients receiving brand-name agents^14–17^. Further analysis showed that our patients had similar SVR_12_ rates to the phase II and III clinical trials by cirrhosis or genotype/subtype status^14–17,45^. Furthermore, the SVR_12_ rates in our patients receiving SOF/LDV-based therapy were comparable irrespective of baseline patient characteristics, implying the use of generic SOF/LDV can also achieve excellent effectiveness.

About 66.5% of our patients were treated by generic SOF/DCV or SOF/VEL, probably due to the pangenotypic potency and relatively low pill burden compared to SOF/RBV or SOF/LDV^46^. The SVR_12_ rates in our patients receiving generic SOF/DCV and SOF/VEL-based therapies were excellent and were comparable to the phase III clinical trials and real-world reports^18–21,47^. Of 23 decompensated cirrhotic patients receiving generic SOF/DCV or SOF/VEL with RBV for 12 weeks, or SOF/DCV or SOF/VEL without RBV for 24 weeks, all achieved SVR_12_, indicating these agents still had good therapeutic effects in critically ill patients. Furthermore, the response rates remained excellent in patients with unfavorable baseline characteristics. Our data indicated that generic SOF/DCV or SOF/VEL also had similar effectiveness to brand-name agents.

Regarding our 43 decompensated cirrhotic patients, 39 (90.7%) of them achieved SVR_12_ by generic SOF-based DAA therapies. Most patients had improving Child-Pugh class and MELD scores following treatment, implying that the mortality and morbidity can potentially be reduced in these very sick patients.

Among our 32 HBV/HCV coinfected patients not receiving antiviral agents for HBV, the risk of HBV reactivation and the HBV-related hepatitis after generic SOF-based therapies were similar to a recent prospective cohort enrolling 101 HBV/HCV coinfected patients receiving brand-name SOF/LDV, indicating that applying generic DAAs may not increase the risk of HBV reactivation and its associated complication^48^. However, watchful surveillance of HBV activity is still needed to detect and treat HBV-related hepatitis and hepatic decompensation earlier.

Our data showed that the rates of HCV RNA level < LLOD at week 4 of treatment were 88.2–90.3% in patients receiving SOF-based regimens. Although about 10% of our patients remained viremic at week 4 of treatment, the SVR_12_ rates were comparable to those who were aviremic at week 4 of treatment. Therefore, the early virokinetics plays a minor role in predicting SVR_12_ in patients receiving generic SOF-based therapies^49,50^. Furthermore, our data was also in accordance with a recent meta-analysis that the SVR_12_ rates of SOF-based therapies were comparable in Asian patients with or without history of HCC^51^.

In addition to the excellent safety profiles and effectiveness, the prices of generic SOF-based DAA therapies are about 1–2% of the brand-name agents^52^. Based on these advantages, the use of generic SOF-based IFN-free DAA regimens may facilitate the mass treatment and play an important role in the elimination of HCV infection in the world, particularly in resource-constrained countries^53^. However, prudential assessment of severity of hepatic fibrosis, particularly for hepatic decompensation, and HCV genotype are still needed to optimize the treatment strategies.

Although generic SOF-based IFN-free DAAs had excellent safety and effectiveness, several limitations existed in our study. First, we included patients with different characteristics and the direct comparison of effectiveness and safety for each SOF-based regimen was not feasible. Second, the generic DAAs were made by various pharmaceutical companies and the direct comparison of effectiveness and safety was difficult. Third, data regarding the on-treatment constitutional or laboratory adverse events were not available in our retrospective study, making the detailed safety analysis impossible.

In summary, generic SOF-based IFN-free regimens achieved comparably excellent effectiveness and safety to the brand-name agents. These regimens may improve the care of HCV for patients with limited access to the expensive brand-name agents.
